# A small molecule transcription factor EB activator ameliorates beta‐amyloid precursor protein and Tau pathology in Alzheimer's disease models

**DOI:** 10.1111/acel.13069

**Published:** 2019-12-19

**Authors:** Ju‐Xian Song, Sandeep Malampati, Yu Zeng, Siva Sundara Kumar Durairajan, Chuan‐Bin Yang, Benjamin Chun‐Kit Tong, Ashok Iyaswamy, Wen‐Bin Shang, Sravan Gopalkrishnashetty Sreenivasmurthy, Zhou Zhu, King‐Ho Cheung, Jia‐Hong Lu, Chunzhi Tang, Nenggui Xu, Min Li

**Affiliations:** ^1^ Medical College of Acupuncture‐Moxibustion and Rehabilitation Guangzhou University of Chinese Medicine Guangzhou China; ^2^ Mr. and Mrs. Ko Chi Ming Centre for Parkinson's Disease Research School of Chinese Medicine Hong Kong Baptist University Hong Kong SAR China; ^3^ Division of Mycobiology & Neurodegenerative Disease Research Department of Microbiology School of Life Sciences Central University of Tamil Nadu Tiruvarur India; ^4^ School of Biomedical Sciences The Chinese University of Hong Kong Hong Kong SAR China; ^5^ State Key Laboratory of Quality Research in Chinese Medicine Institute of Chinese Medical Sciences University of Macau Macao China

**Keywords:** Alzheimer's disease, beta‐amyloid, curcumin analog C1, MAPT/Tau, TFEB

## Abstract

Accumulating studies have suggested that targeting transcription factor EB (TFEB), an essential regulator of autophagy‐lysosomal pathway (ALP), is promising for the treatment of neurodegenerative disorders, including Alzheimer's disease (AD). However, potent and specific small molecule TFEB activators are not available at present. Previously, we identified a novel TFEB activator named curcumin analog C1 which directly binds to and activates TFEB. In this study, we systematically investigated the efficacy of curcumin analog C1 in three AD animal models that represent beta‐amyloid precursor protein (APP) pathology (5xFAD mice), tauopathy (P301S mice) and the APP/Tau combined pathology (3xTg‐AD mice). We found that C1 efficiently activated TFEB, enhanced autophagy and lysosomal activity, and reduced APP, APP C‐terminal fragments (CTF‐β/α), β‐amyloid peptides and Tau aggregates in these models accompanied by improved synaptic and cognitive function. Knockdown of TFEB and inhibition of lysosomal activity significantly inhibited the effects of C1 on APP and Tau degradation in vitro. In summary, curcumin analog C1 is a potent TFEB activator with promise for the prevention or treatment of AD.

## Graphical abstract

A small molecule activator of transcription factor EB (TFEB) promotes the degradation of beta‐amyloid precursor protein (APP) fragments, β‐amyloid peptides (Aβ), and phosphorylated MAPT/Tau aggregates in three transgenic mice models of Alzheimer's disease (P301S, 5xFAD, and 3xTg) and prevents memory impairments.

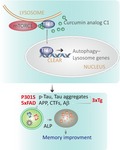

**Abbreviations**
AD: Alzheimer's disease
ALP: autophagy–lysosome pathway
APP: beta‐amyloid precursor protein
Aβ: β‐amyloid peptides
BACE1: beta‐secretase 1
BBB: blood–brain barrier
CMC‐Na: sodium carbonyl methylcellulose
CTFs: C‐terminal fragments
CTSD: cathepsin D
LAMP1: lysosomal‐associated membrane protein 1
MAP1LC3B/LC3B: microtubule‐associated protein 1 light chain 3B
MAPK1: mitogen‐activated protein kinase 1
MAPT: microtubule‐associated protein tau
MTORC1: rapamycin (serine/threonine kinase) complex 1
NFT: neurofibrillary tangles
PSEN1: presenilin 1
RPS6KB1/p70S6K: ribosomal protein S6 kinase, polypeptide 1
SQSTM1/p62: sequestosome 1
TFEB: transcription factor EB

## 1 INTRODUCTION

Alzheimer's disease (AD) is the most prevalent neurodegenerative disease which causes dementia in the elderly. The main pathological features of AD are extracellular amyloid plaques, composed of β‐amyloid peptides (Aβ), and intracellular neurofibrillary tangles (NFT), composed of hyperphosphorylated MAPT/Tau protein, in brains. The autophagy–lysosome pathway (ALP) is a highly efficient process for degrading and recycling cellular components including toxic protein aggregates and damaged organelles. Increasing evidence has indicated that the turnover of beta‐amyloid precursor protein (APP), Aβ metabolism (Nixon, 2007; Pickford et al., 2008; Yang et al., 2011) and MAPT protein degradation (Kruger, Wang, Kumar, & Mandelkow, 2012; Schaeffer et al., 2012; Wang et al., 2009) are mainly regulated by ALP, and ALP impairment plays important roles in AD pathogenesis (Nixon & Yang, 2011; Orr & Oddo, 2013).

The discovery of transcription factor EB (TFEB) as a master regulator of ALP (Sardiello et al., 2009; Settembre et al., 2011) has led to a series of translational studies aiming to explore the therapeutic potential of targeting TFEB for treating human diseases, especially neurodegenerative disorders involving autophagy–lysosomal dysfunction (Ballabio, 2016). Specific to AD, overexpression of TFEB has been shown to be capable of attenuating APP/Aβ or MAPT pathology. Exogenous TFEB expression mediated by AAV injection in hippocampal astrocytes and neurons of APP/PS1 mice reportedly promotes the degradation of APP and APP C‐terminal fragments (CTFs) and reduces Aβ generation (Xiao et al., 2014, 2015). In tauopathy mice, TFEB overexpression mediated by AAV delivery or transgene targeted hyperphosphorylated and misfolded MAPT for degradation (Polito et al., 2014), reversed learning deficits (Wang, Wang, Carrera, Xu, & Lakshmana, 2016) and reduced MAPT spreading (Martini‐Stoica et al., 2018). These findings indicate that TFEB is a promising drug target for AD treatment. However, all the above‐mentioned studies were based on direct TFEB gene transfer into the brain. Furthermore, whether TFEB activation can be protective in AD mice overexpressing both APP and MAPT has not been investigated. Small molecule activators of TFEB have seldom been discovered, and their neuroprotective effects in AD animal models have not been fully evaluated.

In searching for new TFEB activators, we previously identified a novel curcumin analog C1, which specifically binds to and activate TFEB without inhibiting mechanistic target of rapamycin (serine/threonine kinase) complex 1 (MTORC1) and mitogen‐activated protein kinase 1 (MAPK1/ERK2) activity (Song et al., 2016). Notably, this small molecule passes easily through the blood–brain barrier (BBB). Oral administration of curcumin analog C1 activated TFEB and promoted autophagy–lysosome biogenesis in mice brains (Song et al., 2016). In this study, we investigated the efficacy of curcumin analog C1 in three AD animal models, which represent the APP pathology (5xFAD mice), tauopathy (P301S Tau mice) and the APP/MAPT combined pathology (3xTg‐AD mice). In these models, curcumin analog C1 activated TFEB, promoted autophagy and lysosome biogenesis, degraded APP and Tau aggregates, reduced the levels of Aβ, reversed synaptic dysfunction and improved cognitive deficits. Furthermore, we showed that TFEB is required for the autophagic degradation of APP and Tau by compound C1 in vitro.

## 2 RESULTS

### 2.1 Curcumin analog C1 activates TFEB, promotes the degradation of phosphorylated Tau aggregates and improves motor function in P301S mice

Previous studies have shown that TFEB overexpression can remarkably reduce the hyperphosphorylated and misfolded MAPT/Tau proteins, and rescue behavioral/learning deficits in rTg4510 (Polito et al., 2014) and P301S (Wang et al., 2016) mice models of tauopathy. Here, we investigated whether a small molecule TFEB activator C1 (Song et al., 2016) discovered by us could be as effective as TFEB overexpression in attenuating tauopathy by using homozygous P301S Tau mice developed by Michael Goedert (Allen et al., 2002). Since no gender differences on pathology have been reported in P301S mice, we combined both male and female mice in our study. One‐month‐old P301S mice received oral treatment of compound C1 for 3 month, and then, Rotarod test was performed (Figure S1A) to evaluate the balance and coordination, which has prominent deficit in P301S mice at 4 months of age (Scattoni et al., 2010). The result showed that mice treated with C1 spent longer time on the accelerating rotarod compared with vehicle‐treated mice (Figure S1B). Then, we determined the levels of phosphorylated (p‐) Tau AT8 (S202/T205), PHF1 (S396/S404) and total Tau in the brain lysates extracted by RIPA buffer (Figure S1C,D). The quantification data in Figure S1E,F, demonstrate that the ratio of AT8 and PHF1 to total Tau was significantly reduced in C1 treatment group.

**Figure 1:**
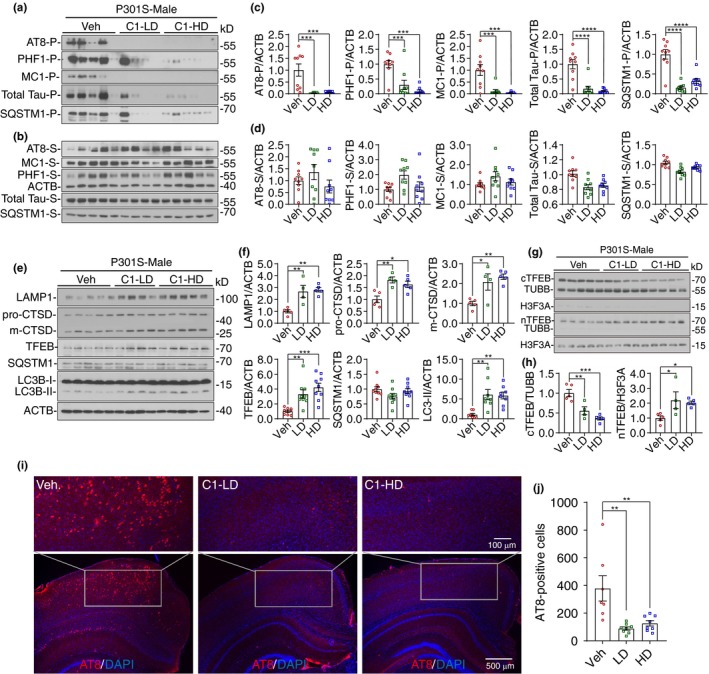
Curcumin analog C1 activates transcription factor EB (TFEB), promotes autophagy and lysosome biogenesis, and reduces insoluble Tau aggregates in P301S mice brains. (a, b) Western blots showed the levels of Tau proteins and SQSTM1 in male P301S mice brains (Blots for female mice were shown in Figure S1G). The brain lysates were separated into sarkosyl‐insoluble (P) (a) and sarkosyl‐soluble (S) (b) fractions. (c, d) All the values from sarkosyl‐insoluble (P) (c) and sarkosyl‐soluble (S) (d) fractions were quantified as average ± *SEM* (male and female, *n* = 9). ***p < .001 and ****p < .0001 vs. Veh. group respectively analyzed by one‐way ANOVA. (e) Western blots showed the levels of TFEB, autophagy and lysosome markers in male P301S mice brains (Blots for female mice were shown in Figure S1H). (f) Data were quantified as average ± *SEM*. *p < .05, **p < .01 and ***p < .001 vs. Veh. group respectively analyzed by one‐way ANOVA. (g) Western blots showed the levels of cytosolic and nuclear TFEB in male P301S mice brains. TUBB and H3F3A were used as cytosolic and nuclear controls, respectively. (h) Data were quantified as average ± *SEM* (male, *n* = 4–5). *p < .05, **p < .01, and ***p < .001 vs. Veh. group, respectively, analyzed by one‐way ANOVA. (i) Representative immunofluorescence staining of AT8‐positive cells in the cortex and hippocampus of male P301S mice brains. (j) All the values from were quantified as average ± *SEM* (male and female, *n* = 6–8). **p < .01 vs. Veh. group analyzed by one‐way ANOVA

Next, we determined whether C1 reduces soluble or insoluble Tau species by sarkosyl fractioning of mice brains (Figure 1a,b). Notably, we found that C1 treatment drastically reduced the levels of p‐Tau (AT8 and PHF1), conformation‐specific Tau (MC1), and total Tau in the sarkosyl‐insoluble (P) fractions (Figure 1c), and the effects are comparable to that of TFEB overexpression as previously investigated (Polito et al., 2014). Importantly, the levels of SQSTM1, an autophagy substrate, were also dramatically reduced by C1 treatment in the sarkosyl‐insoluble fractions (Figure 1c). In contrast, the levels of AT8, PHF1, MC1, total Tau, and SQSTM1 were not significantly decreased by C1 treatment in the sarkosyl‐soluble (S) fractions (Figure 1d). The immunofluorescence staining results of AT8 in the cortex and hippocampus further validated that C1 treatment drastically reduced the phosphorylated Tau (Figure 1i,j). These results indicate that C1 mainly reduces insoluble Tau aggregates in P301S mice brains.

To find out whether the reduction in Tau aggregates by C1 is mediated by TFEB, we simultaneously determined the levels of TFEB, the cytosolic/nuclear TFEB, the autophagy markers (LC3B and SQSTM1) and the lysosome markers (LAMP1 and CTSD) in the brain lysates (Figure 1e–h; Figure S1G,H). The results showed that C1 not only increased the levels of total TFEB, but also promoted the nuclear translocation of TFEB (Figure 1h). For the autophagy markers, C1 treatment significantly increased the levels of LC3B‐II, while leaving SQSTM1 unchanged in the RIPA‐soluble fractions (Figure 1f). For lysosome markers, C1 treatment significantly increased the levels of LMAP1 and pro‐/mature‐CTSD (Figure 1f), which are target proteins regulated by TFEB. Notably, C1 treatment significantly activated the MTOR pathway, as indicated by the increase in the levels of phosphorylated (p‐) MTOR and its substrate ribosomal protein S6 kinase, polypeptide 1 (RPS6KB1/p70S6K) (Figure S1I,J). These results indicate that C1 treatment activates TFEB and promotes autophagy and lysosome biogenesis in the brain of P301S mice without inhibiting the MTOR pathway.

### 2.2 Curcumin analog C1 activates TFEB, degrades APP/Aβ, and prevents synaptic and cognitive failures in 5xFAD mice

**Figure 2:**
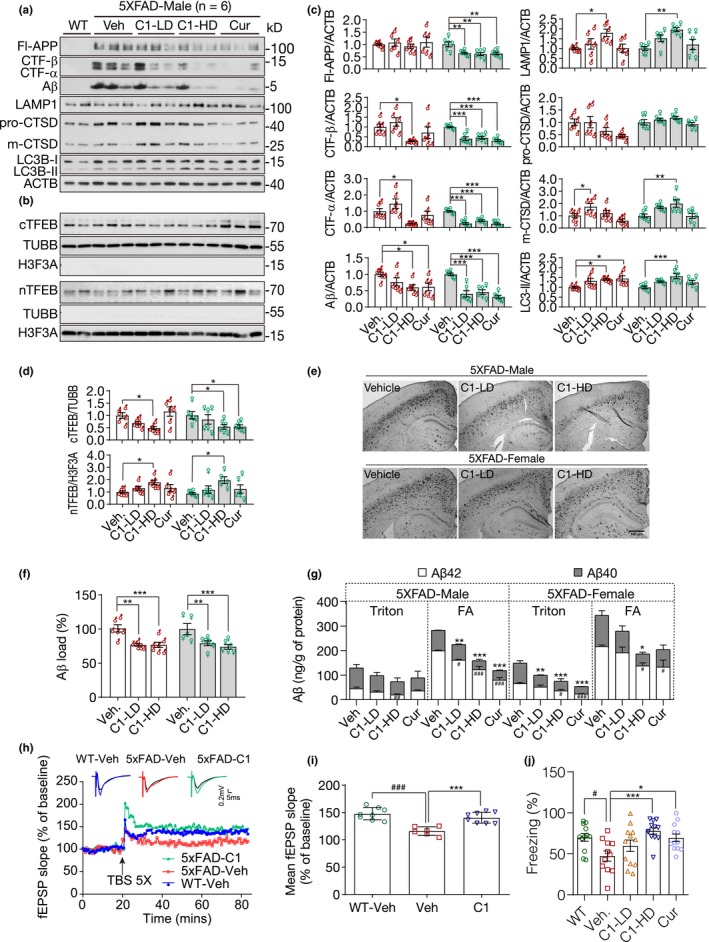
Curcumin analog C1 activates transcription factor EB (TFEB), degrades beta‐amyloid precursor protein (APP) and β‐amyloid peptides (Aβ), and prevents synaptic and cognitive failures in 5xFAD mice. (a, b) Western blots showed the levels of full‐length APP (Fl‐APP), CTF‐β/α, Aβ, LAMP1, CTSD, LC3B, cytosolic and nuclear TFEB (cTFEB and nTFEB) with their respective loading controls in the brain lysates of male mice (Another batch of blots for male mice and all blots for female mice were shown in Figure S2B). (c, d) All the values from male (*n* = 6) and female (*n* = 6) mice were quantified as average ± *SEM*. *p < .05, **p < .01, and ***p < .001 vs. Veh. group, respectively, analyzed by one‐way ANOVA. The interaction between sex and drug effects was analyzed by two‐way ANOVA, and the P values were summarized in Table S1. (e, f) Immunohistochemistry analysis of Aβ load. (e) Representative images show Aβ labeled with biotinylated 4G8 antibody. (f) Data are quantified as mean ± *SEM* in male (*n* = 6) and female (*n* = 6) mice. **p < .01 and ***p < .001 vs. Veh. group, respectively. (g) ELISA assay of Aβ42 and Aβ40 in male (*n* = 6) and female (*n* = 6) mice. Whole‐brain samples were separated into Triton soluble and formic acid (FA) fractions. Data were presented as mean ± *SEM*. ^#^ *p* < .05, ^##^ *p* < .01, and ^###^ *p* < .001 vs. Veh. group (Aβ42); *p < .05, **p < .01, and ***p < .001 vs. Veh. group (Aβ40). The interaction between sex and drug effects was analyzed by two‐way ANOVA, and the *p* values were summarized in Table S1. (h, i) C1 restores synaptic plasticity in female 5xFAD mice. (h) Representative time course of fEPSP slope recorded in WT‐Veh, 5xFAD‐Veh, and 5xFAD‐C1 groups before and after TBS stimulation. Traces were normalized and represented as percentage of baseline response. Individual EPSP response traces before (black) and after TBS stimulation (colored) were shown in the above as indicated. (i) Mean percentage of fEPSP slope recorded in WT, 5xFAD‐Veh and 5xFAD‐C1 groups (*n* = 6). One‐way ANOVA: ^###^ *p* < .001 vs. WT‐Veh; ***p < .001 vs. 5xFAD‐Veh. (j) Contextual fear conditioning test. The percentage of freezing in each group was quantified as mean ± *SEM* (*n* = 12, both sex). ^#^ *p* < .05 vs. wild‐type (WT) mice; *p < .05, **p < .01, and ***p < .001 vs. Veh. group, respectively, analyzed by one‐way ANOVA. LAMP, lysosomal‐associated membrane protein 1

According to previous studies, TFEB overexpression is not only effective on attenuating tauopathy but can also attenuate amyloid pathogenesis by reducing APP and Aβ (Xiao et al., 2014, 2015). Therefore, we evaluated the effects of curcumin analog C1 on TFEB activation, APP/Aβ degradation, synaptic dysfunction and memory deficits in 5xFAD mice. Since curcumin has been previously reported to be protective in 5xFAD mice (McClure et al., 2017; Zheng et al., 2017), we used it as a parallel control in this study. The experimental protocol is shown in Figure S2A. Western blot analysis showed that C1 treatment significantly reduced the levels of full‐length APP (Fl‐APP), CTF‐α/β and Aβ, accompanied by TFEB activation, increased autophagy (indicated by LC3B‐II) and lysosome activity (indicated by LAMP1 and CTSD) in 5xFAD mice (Figure 2a–d; Figure S2B). The effects of C1 on Aβ load were further determined by immunohistochemistry and ELISA assay, and the quantification results showed significant reduction of Aβ load in cortico‐hippocampal sections (Figure 2e,f) and reduced levels of Aβ_42_ and Aβ_40_ in whole‐brain lysates (Figure 2g) from both male and female mice treated with C1.

Whether gender affects the drug effects was analyzed by two‐way ANOVA (Table S1). The results showed that sex tends to significantly affect the drug effects on APP, CTF‐α/β, and Aβ. No significant sex effects were observed on CTSD, LAMP1, TFEB, and LC3B. However, after a careful examination of the data, we found that the sex differences in APP, CTF‐α/β, and Aβ are mainly attributed by the C1 (LD) group. Significant variations were observed for the effects of C1 (LD) on APP, CTF‐α/β, and Aβ in male and female mice. If the analysis is only performed on the vehicle and C1 (HD) groups, no significant sex difference was found. The results indicate that the ability of C1 on TFEB activation and the ALP machinery may be similar in both male and female mice. However, since female 5xFAD mice have more severe Aβ pathology (Devi, Alldred, Ginsberg, & Ohno, 2010), they tend to be more sensitive to sub‐effective dose of C1. Our result is consistent with the findings of Xiao et al. (2015), which showed no gender difference for TFEB overexpression on reduction of APP, CTF‐α/β, and Aβ load in APP/PS1 mice.

Curcumin treatment at 50 mg/kg showed similar effects as C1 (HD) on reducing Fl‐APP, CTF‐α/β, and Aβ. However, when comparing their effects on TFEB‐mediated autophagy and lysosome biogenesis, compound C1 showed much better activity than curcumin. Curcumin only has mild but not significant effects on increasing TFEB nuclear translocation and the levels of LAMP1, CTSD, and LC3B‐II (Figure 2c). On the other hand, curcumin, but not C1, significantly inhibited MTORC1 activity evidenced by the decrease in p‐MTOR and its substrate p‐RPS6KB1 in the brains of 5xFAD mice (Figure S2C,D). These results indicate that although curcumin can reduce APP and Aβ at a relatively high dose, its effect may be mainly mediated by MTORC1 inhibition or BACE1 inhibition as reported previously (Zheng et al., 2017).

In our previous study, we had shown that C1 could pass the blood–brain barrier (BBB) in healthy rats (Song et al., 2016). To further support the efficacy of C1 in 5xFAD mice, we determined the concentration of compound C1 in the brains of 5xFAD mice. The concentration of C1 was 216.7 ± 55.3 ng/g (equivalent to 0.74 ± 0.19 μM) and 261.2 ± 32.9 ng/g (equivalent to 0.89 ± 0.11 μM) in male (*n* = 5) and female (*n* = 5) mice, respectively (Figure S2E). Together, these results indicate that C1 promotes the degradation of APP fragments and reduces Aβ by promoting TFEB‐mediated autophagy and lysosomal biogenesis in the brains of 5xFAD mice.

To determine whether C1‐induced TFEB activation and degradation of APP/Aβ improves synaptic and cognitive function in 5xFAD mice, we performed long‐term potentiation (LTP) recording and contextual fear conditioning test, which were deficient in 5xFAD mice at 6 months of age (Kimura & Ohno, 2009). The LTP at the Schaffer collateral‐CA1 synapses was significantly reduced in 6‐month‐old vehicle‐treated 5xFAD mice compared with age‐matched vehicle‐treated WT mice (WT‐Veh vs. 5xFAD‐Veh, *p* < .001). 5xFAD mice treated with C1 for 3 months showed significant restoration of LTP (5xFAD‐C1 vs. 5xFAD‐Veh, *p* < .001; Figure 2h,i). Compared with WT mice, the freezing time of 5xFAD mice significantly decreased. C1 (HD) and curcumin‐treated (50 mg/kg) mice showed significant memory reconsolidation evidenced by the increased freezing index compared with the vehicle treatment (Figure 2j). C1 showed better memory improvement than curcumin in 5xFAD mice.

### 2.3 Curcumin analog C1 attenuates both APP and Tau pathology and improves cognitive deficits in 3xTg‐AD mice

**Figure 3:**
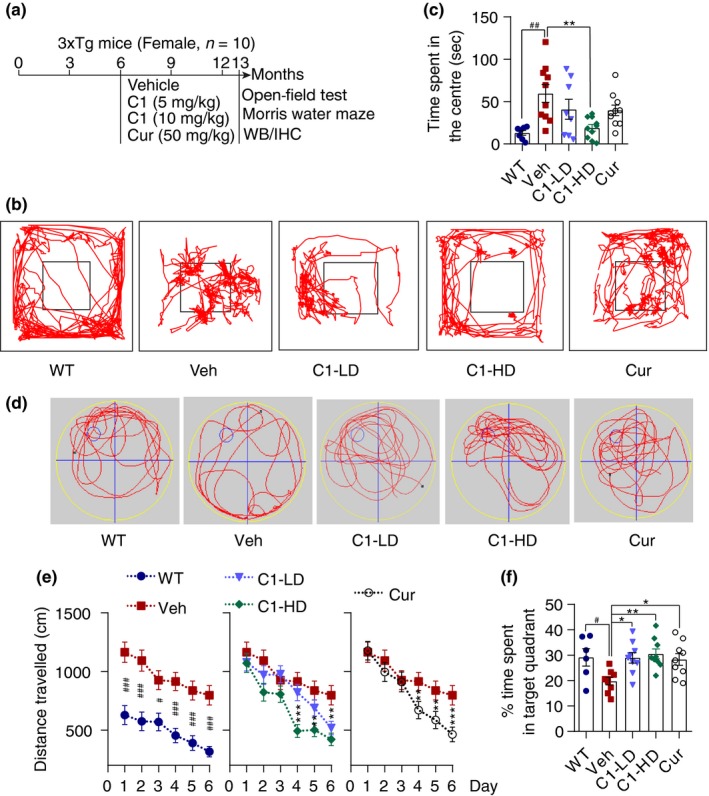
Curcumin analog C1 improves exploratory behavior, spatial learning, and memory acquisition in female 3xTg mice. (a) Experimental protocols for female 3xTg mice. (b) Open‐field test. Representative exploratory patterns of mice in each group. (c) Quantification of time spent in the center for each treatment (mean ± *SEM*; *n* = 10). ^##^ *p* < .01 vs. WT; **p < .01 vs. Veh. treatment analyzed by one‐way ANOVA. (d) Morris water maze. Representative moving patterns of mice in each group in the probe trials. (e) Quantification of the distance travelled (mean ± *SEM*) to find the hidden platform (*n* = 10). ^#^ *p* < .05, ^###^ *p* < .001 vs. WT; **p < .01, ***p < .001 vs. Veh. treatment analyzed by one‐way ANOVA. (f) Quantification of time spent in the target quadrant (mean ± *SEM*) for each treatment group (*n* = 10) in the probe trials. ^#^ *p* < .05 vs. WT; *p < .05, **p < .01 Veh. treatment analyzed by one‐way ANOVA

Previous studies had evaluated the protective effects of TFEB overexpression in APP or Tau transgenic mice of AD. However, whether TFEB overexpression/activation by gene transfer or small molecule activators could be neuroprotective in 3xTg mice which expresses both APP and Tau has not been investigated. Therefore, we evaluated the efficacy of curcumin analog C1 on improving cognitive function and degradation of APP/Tau products in 3xTg mice. Since male 3xTg mice may not exhibit the phenotypic traits as reported by the donating investigator, only female 3xTg mice were used in this study. Six‐month‐old female 3xTg mice were treated with C1 or curcumin for 7 months. The open‐field test and Morris water maze were applied to evaluate memory improvement and molecular changes in APP, Tau, TFEB and autophagy were determined (Figure 3a).

In the open‐field test, the 3xTg mice stayed much longer in the center compared with the WT mice (*p* < .001; Figure 3c). In contrast, C1 treatment dose‐dependently decreased the time spent in the center compared with vehicle treatment. The 3xTg mice treated with the high dose of C1 (10 mg/kg) almost recovered exploratory behavior to the levels of WT mice (*p* < .001; Figure 3b). However, curcumin treatment showed no significant improvement for 3xTg mice in the open‐field test.

In the Morris water maze test, the 3xTg mice treated with C1 (10 mg/kg) travelled a significantly shorter distance to find the hidden platform (Figure 3e) compared with vehicle treatment. In probe trial, the 3xTg mice treated with C1 (5, 10 mg/kg) or curcumin (50 mg/kg) significantly spent a significantly greater percentage of time in the target quadrant when compared to the vehicle treatment (Figure 3d,f). Notably, 3xTg mice treated with C1 (10 mg/kg) showed much better improvement in target recognition than those treated with curcumin (50 mg/kg). These results indicate C1 ameliorates spatial learning and memory impairment in 3xTg AD mice.

**Figure 4:**
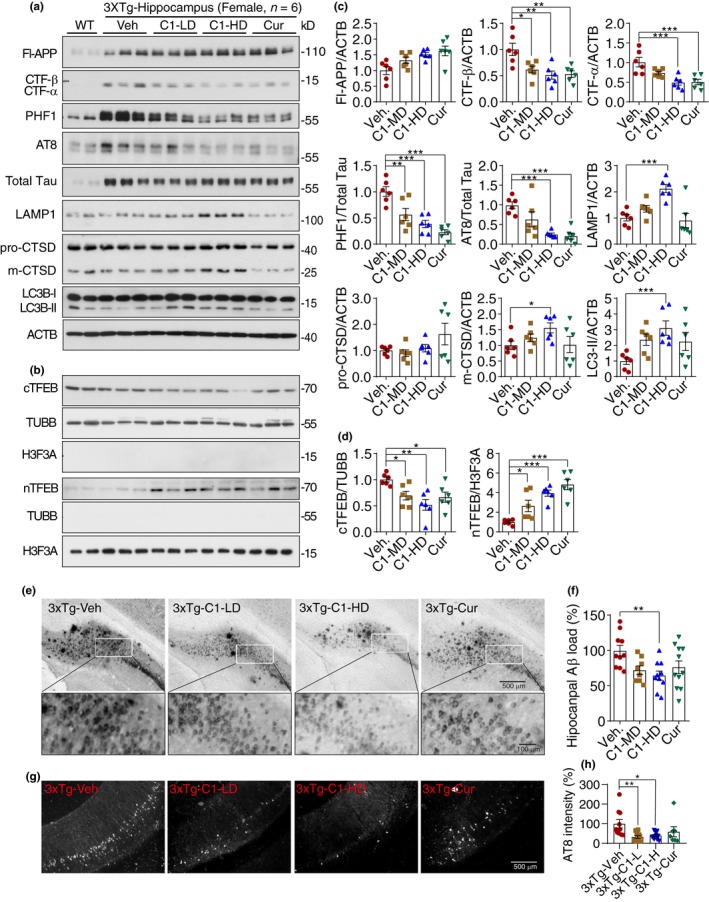
Curcumin analog C1 activates transcription factor EB (TFEB) and attenuates both beta‐amyloid precursor protein (APP) and Tau pathology in 3xTg mice. (a, b) Representative blots showed the levels of Fl‐APP, CTF‐β/α, phosphorylated Tau (PHF‐1, AT‐8), total Tau, LAMP1, CTSD, cytosolic and nuclear TFEB (cTFEB and nTFEB), and LC3B with their respective loading controls in the hippocampus of female mice (Another batch of blots were shown in Figure S3A). (c, d) Data are quantified as mean ± *SEM* (*n* = 6). *p < .05, **p < .01, ***p < .001 vs. Veh. treatment analyzed by one‐way ANOVA. (e, f) Immunohistochemistry analysis of β‐amyloid peptides (Aβ) load. (e) Representative images show the Aβ labeled with biotinylated 4G8 antibody. (f) Data are quantified as mean ± *SEM* (*n* = 10). *p < .05 vs. Veh. treatment analyzed by one‐way ANOVA. (g, h) Immunohistochemistry analysis of p‐Tau (AT8). (g) Representative images show AT8 staining in the posterior hippocampus. (h) Data are quantified as mean ± *SEM* (*n* = 7–12). *p < .05, **p < .01 vs. Veh. treatment analyzed by one‐way ANOVA. CTSD, cathepsin D; LAMP, lysosomal‐associated membrane protein 1

To determine whether the effects of C1 on memory improvement in 3xTg mice can be attributed by its ability to attenuate APP and Tau pathology, the levels of different forms of APP and Tau in the hippocampus of female mice were semi‐quantified by immunoblots. As shown in Figure 4, the overexpression of APP and Tau in 3xTg mice was verified in comparison with the WT mice. For APP and Aβ, C1 high dose (10 mg/kg) significantly reduced hippocampal CTF‐α/β, but not Fl‐APP, levels (Figure 4c; Figure S3A) and reduced Aβ load in the hippocampus (Figure 4e,f). For Tau species, both C1 (10 mg/kg) and curcumin (50 mg/kg) treatment significantly decreased the levels of phosphorylated Tau (PHF1, AT8), but not the total Tau (Figure 4c; Figure S3A). Meanwhile, C1 (10 mg/kg) significantly promoted TFEB nuclear translocation (Figure 4b,d; Figure S3A) and increased LC3B‐II, LAMP1 and mature‐CTSD in the hippocampus (Figure 4c; Figure S3A) without inhibiting MTORC1 activity (Figure S3B,C). Notably, we found that C1 treatment has no significant effects on the levels of endogenous Fl‐APP, CTF‐α/β, PHF1, and total Tau in the brains of WT mice (Figure S4). These results indicate that C1 efficiently promotes the degradation of APP fragments, Aβ and phosphorylated Tau aggregates in 3xTg‐AD mice by promoting TFEB‐mediated autophagy and lysosomal biogenesis. In comparison with C1, curcumin at 50 mg/kg showed similar effects as C1 (HD) on reducing CTF‐α/β and p‐Tau. However, curcumin showed no significant effects on the levels of LAMP1, CTSD, LC3B‐II (Figure 4c) and Aβ load (Figure 4f).

### 2.4 TFEB is required for compound C1 to degrade APP and MAPT/Tau in vitro

**Figure 5:**
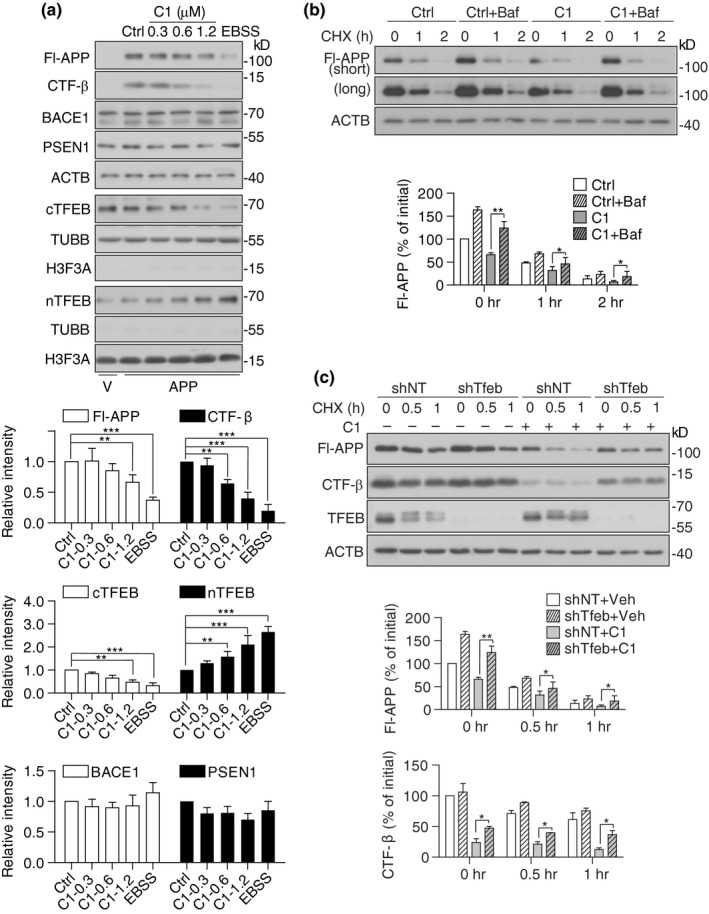
Transcription factor EB (TFEB) is required for compound C1 to degrade beta‐amyloid precursor protein (APP) and CTFs in vitro. (a) N2a cells were transfected with APPSwe/Ind plasmid or empty vector (V) for 24 hr and then treated with C1 (0–1.2 μM) for 16 hr. EBSS treatment for 4 hr was used as a positive control. Data are presented as the average ± *SEM* from three independent experiments. *p < .05, **p < .01, ***p < .001 vs. DMSO control. (b) N2a cells expressing APP were pretreated with C1 (1.2 μM) for 14 hr. bafilomycin A1 (Baf, 100 nM) was added into the cells for 30 min and then co‐treated with cycloheximide (CHX, 50 μg/ml) for the indicated times. Data are presented as the average ± *SEM* from three independent experiments. *p < .05 and **p < .01 vs. C1 treatment alone at each time point. (c) N2a cells with/without stable Tfeb knockdown were transfected with APPSwe/Ind plasmid for 24 hr and then treated with C1 (1.2 μM) for 14 hr. Cycloheximide (CHX, 50 μg/ml) was added, and the cells were collected at the indicated time points. Data are presented as the average ± *SEM* from three independent experiments. *p < .05 and **p < .01 vs. C1 treatment alone at each time point

**Figure 6:**
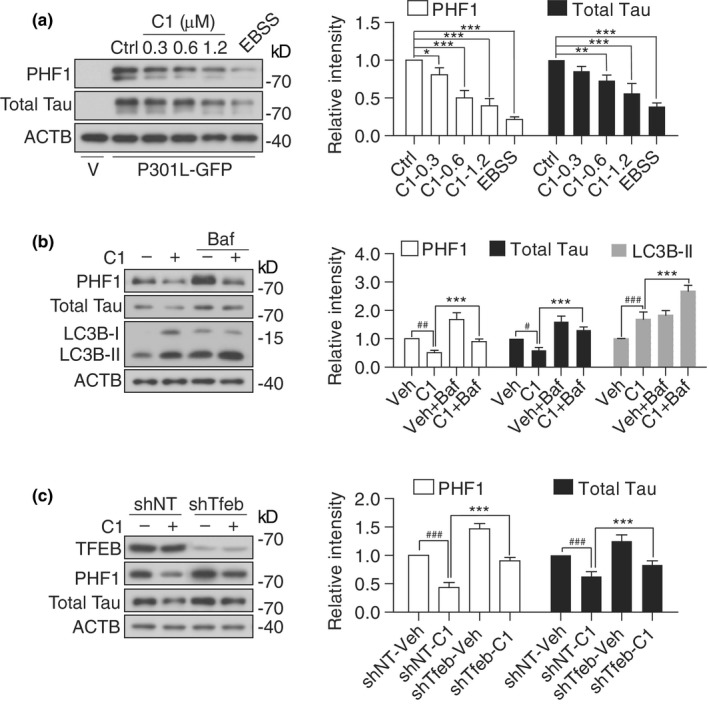
Transcription factor EB (TFEB) is required for compound C1 to degrade MAPT/Tau in vitro. (a) N2a cells were transfected with P301L‐Tau plasmid or empty vector (V) for 24 hr and then treated with C1 (0–1.2 μM) for 16 hr. EBSS treatment for 4 hr was used as a positive control. Data are presented as the average ± *SEM* from three independent experiments. *p < .05, **p < .01, ***p < .001 vs. DMSO control. (b) N2a cells expressing P301L‐Tau were pretreated with C1 (1.2 μM) for 12 hr and then co‐treated with bafilomycin A1 (Baf, 100 nM) for an additional 4 hr. Data are presented as the average ± *SEM* from three independent experiments. ^#^ *p* < .05, ^##^ *p* < .01, ^###^ *p* < .001 vs. vehicle control (DMSO); *p < .05, **p < .01, ***p < .001 vs. C1 treatment alone. (c) N2a cells with/without stable Tfeb knockdown were transfected with P301L‐Tau plasmid for 24 hr and then treated with C1 (1.2 μM) for 16 hr. Data are presented as the average ± *SEM* from three independent experiments. ^###^ *p* < .001 vs. shNT‐Veh; ***p < .001 vs. shNT‐C1

Our animal studies have demonstrated the efficacy of compound C1 on reducing APP CTF‐α/β, Aβ and p‐Tau aggregates in vivo, accompanied by TFEB activation, enhanced autophagy and lysosomal activity. To further validate that the anti‐AD efficacy of C1 is mainly mediated by TFEB, we tested the effects of C1 on APP turnover and Tau degradation in neuronal cell cultures. Firstly, in N2a cells overexpressing APP695, C1 dose‐dependently promoted the nuclear translocation of TFEB and reduced the levels of Fl‐APP and CTF‐β detected by 6E10 antibody (Tian, Bustos, Flajolet, & Greengard, 2011; Figure 5a). Earle's balanced salt solution (EBSS) starvation for 4 hr was used as a positive control. Notably, C1 did not affect the expression of BACE1/beta‐secretase 1 and PSEN1/presenilin 1 (one major component of γ‐secretase), which are involved in the amyloidogenic processing of APP. To determine whether C1 promotes the lysosomal degradation of APP, we used bafilomycin A1 (Baf) to inhibit lysosomal acidification and cycloheximide (CHX) to inhibit protein synthesis. The effects of C1 on APP reduction were significantly inhibited by Baf at each indicated time point of CHX treatment (Figure 5b). Furthermore, the effect of C1 on APP/CTF‐β degradation was significantly inhibited in TFEB knockdown cells (Figure 5c). Similar results were observed in N2a cells expressing P301L‐Tau. C1 significantly reduced the levels of PHF1 and total Tau (Figure 6a), and its effects were inhibited by lysosomal inhibition (Figure 6b) and TFEB knockdown (Figure 6c). Together, these results demonstrated that C1 promotes the lysosomal degradation of APP/CTF‐β and Tau via TFEB activation.

### 2.5 GSK3B and AKT are not involved in compound C1‐induced TFEB activation

We previously confirmed that C1 activates TFEB without inhibiting the activities of MTORC1 and MAPK1/ERK2 (Song et al., 2016). Since GSK3B (Marchand, Arsenault, Raymond‐Fleury, Boisvert, & Boucher, 2015) and AKT (Palmieri et al., 2017) had been identified as negative regulators of TFEB, we further determined whether they are involved in C1‐induced TFEB activation. As shown in Figure S5, trehalose reduced p‐AKT (S473), but not p‐GSK3B (S9), which was consistent with the previous finding showing trehalose activated TFEB by inhibiting AKT independently of GSK3B (Palmieri et al., 2017). The GSK3B inhibitor lithium chloride (LiCl) increased both p‐GSK3B (S9) and p‐AKT (S473), which was consistent with previous findings (Chalecka‐Franaszek & Chuang, 1999; Marchand et al., 2015). Compound C1 increased p‐AKT (S473), but not p‐GSK3B (S9), indicating that C1 activates AKT without inhibiting GSK3B. Together, our results indicate that GSK3B and AKT are not involved in C1‐induced TFEB activation.

## 3 DISCUSSION

Accumulating evidence has suggested that targeting TFEB to regulate ALP is a promising approach to developing new drugs to treat neurodegenerative disorders including AD (Chandra, Jana, & Pahan, 2018; Chauhan et al., 2015; Martini‐Stoica et al., 2018; Polito et al., 2014; Wang et al., 2016; Xiao et al., 2014, 2015). However, most of these studies were at the stage of proof‐of‐concept by exogenously overexpressing TFEB. Small molecules which specifically activate TFEB and have good bioavailability are still not available at present. Previously, we identified a curcumin analog C1 as a TFEB activator. The small molecule activates TFEB without inhibiting MTORC1 and ERK2 activity. Notably, the compound directly binds to recombinant TFEB protein, which may indicate that C1 is a direct activator of TFEB. Importantly, the compound has good brain permeability and activates TFEB in vivo (Song et al., 2016). Therefore, in this study we systematically evaluated the protective effects of C1 in AD animal models and demonstrate that curcumin analog C1 can activate TFEB, promote autophagy and lysosomal activity, attenuate Aβ and Tau pathology, and prevent memory impairment in AD. The in vitro studies further proved that TFEB is required for C1 to degrade APP CTFs and Tau. However, whether TFEB is the primary target for C1 to exert anti‐AD efficacy in animal models needs to be further validated.

In our previous study (Song et al., 2016), we confirmed that curcumin analog C1 activates TFEB by directly binding to TFEB and promotes its entry into the nucleus, without affecting TFEB phosphorylation or inhibiting the activities of MTORC1 and MAPK1/ERK2. Furthermore, we demonstrated that GSK3B and AKT are not involved in compound C1‐induced TFEB activation (Figure S5). In addition, MAP4K3 was identified as another negative regulator of TFEB, acting by phosphorylating TFEB on serine 3 (Hsu et al., 2018). Since compound C1 did not affect the total levels of phosphoserine on TFEB (Song et al., 2016), MAP4K3 is not likely involved in C1‐induced TFEB activation, although not determined in this study. Notably, a recent study demonstrated the neurotrophic effects of TFEB overexpression by promoting pro‐survival pathways and protein synthesis, evidenced by AKT activation and phosphorylation of mTORC1 effectors (EIF4EBP1 and RPS6KB1; Torra et al., 2018). Since compound C1 also can activate mTORC1 (Song et al., 2016; Figure S1J) and AKT (Figure S5), the pro‐survival signaling triggered by TFEB activation may also contribute to the neuroprotective effects of C1 in AD models.

Regarding the efficacy and specificity of TFEB overexpression on Tau degradation and APP turnover, previous studies demonstrated somewhat different results. Polito et al. (2014), showed that TFEB overexpression mainly attenuates tauopathy without affecting Aβ deposition. However, the findings from Xiao et al. (2014, 2015) indicated that TFEB overexpression also affects APP turnover and Aβ clearance. In our study, when comparing the results from the P301S Tau mice and 5xFAD mice, C1 has stronger effects on attenuating tauopathy that APP turnover, indicating TFEB activation induced by C1 may primarily act on tauopathy.

Curcumin has been reported to be neuroprotective in various AD models (Potter, 2013); however, its molecular mechanisms are still not fully understood. Whether the anti‐AD efficacy of curcumin is mediated by TFEB activation has not been investigated. As shown in our data, curcumin at a relatively high dose (50 mg/kg) reduced Fl‐APP, CTFs, Aβ, and p‐Tau in 5xFAD and 3xTg mice (Figures 2 and 4). Meanwhile, curcumin inhibited MTORC1 activity in the brains of 5xFAD (Figure S2D) and 3xTg (Figure S3C) mice. However, the results of curcumin on TFEB activation, autophagy, and lysosomal activity were not consistent in 5xFAD and 3xTg mice (Figures 2 and 4). Previously, we have shown that curcumin only had weak effect on TFEB activation in vitro if compared with C1 (Song et al., 2016). Together, our data indicate that curcumin is a weak MTORC1 inhibitor and relatively high dosage of curcumin is needed to activate TFEB and promote autophagy in the brains of AD mice due to the poor absorption, rapid metabolism, and rapid systemic elimination (Anand, Kunnumakkara, Newman, & Aggarwal, 2007).

Mounting evidence has proved that the defects in ALP occur early in the pathogenesis of AD. The progressive disruption of ALP leads to the massive buildup of APP metabolites and Tau aggregates in neurons. Therefore, promoting ALP at the early stage of the disease may be efficient for attenuating the pathology (Nixon & Yang, 2011; Peric & Annaert, 2015; Zare‐Shahabadi, Masliah, Johnson, & Rezaei, 2015). For example, the key autophagy‐related protein BECN1 (beclin‐1) had been reported to be reduced in the brains from early AD patients, and overexpression of BECN1 reduced amyloid pathology in APP mice (Pickford et al., 2008). Another example is that 3xTg mice at 6‐month‐old only present early learning and memory deficits (Billings, Oddo, Green, McGaugh, & LaFerla, 2005). At this stage, administration of an MTORC1 inhibitor rapamycin for 10 weeks could restore mTOR signaling, decrease Aβ and tau pathology, and ameliorate cognitive deficits (Caccamo, Majumder, Richardson, Strong, & Oddo, 2010). A previous study addressing the effects of TFEB overexpression on Tau pathology also used a very early intervention, by injecting TFEB‐AAV construct into the lateral ventricles on postnatal day 0 (P0) of rTg4510 mice brains and analyzed 4 months after (Polito et al., 2014). In our studies, the AD‐Tg mice used were at the early disease stage without overt pathological and cognitive decline, and long‐term treatment with C1 showed drastic improvement. Accordingly, we can only safely conclude that the TFEB activator C1 prevents the disease progression of AD when administered at the early disease stage when the ALP machinery is impaired. Whether overexpressing TFEB or small molecule TFEB activators at late disease stages could reverse the pathology and improve cognition needs to be carefully evaluated in further studies.

## 4 EXPERIMENTAL PROCEDURES

### 4.1 Reagents and antibodies

Curcumin analog C1 was synthesized according to the protocols described in our previous study (Song et al., 2016) with ~98% purity. Curcumin (08511), cycloheximide (CHX, 01810), trehalose (T0167), LiCl (203637), and anti‐SQSTM1/p62 (P0067) were purchased from Sigma‐Aldrich. Anti‐BACE1 (ab108394) antibody was purchased from Abcam. Anti‐H3F3A/histone H3 (D1H2; 4499), anti‐PSEN1 (5643), anti‐phospho‐MTOR (Ser2448) (2971), anti‐MTOR (2983), anti‐phospho‐GSK3B (Ser9) (9336), anti‐GSK3B (12456), anti‐phospho‐AKT (S473), anti‐AKT (9272), and anti‐phospho‐RPS6KB1/P70S6K (Thr389) (9234) antibodies were purchased from Cell Signaling Technology. HRP‐conjugated goat anti‐mouse (115‐035‐003) and goat anti‐rabbit (111‐035‐003) secondary antibodies were purchased from Jackson ImmunoResearch. Anti‐TUBB/β‐tubulin (H‐235) (sc‐9104), anti‐GAPDH (G‐9) (sc‐365062), and anti‐ACTB/β‐actin (sc‐47778) were purchased from Santa Cruz Biotechnology. Anti‐LC3B (NB100‐2220) antibody was purchased from Novus Biologicals. Anti‐TFEB (A303‐673A) was purchased from Bethyl Laboratories, Inc. DMEM (11965‐126), fetal bovine serum (FBS;10270‐106), Opti‐MEM I (31985‐070), anti‐APP (51‐2700), anti‐AT8 (MN1020), Alexa Fluor^®^ 488 goat anti‐mouse IgG (A‐11001), and Alexa Fluor^®^ 594 goat anti‐rabbit IgG (A‐11012) were purchased from Thermo Fisher Scientific. Anti‐β‐Amyloid (6E10) (803017) and biotinylated β‐Amyloid (4G8) (800701) were purchased from Biolegend. Anti‐total Tau (A0024) was purchased from Agilent (Dako). Anti‐PHF1 and MC1 were kindly provided by Prof. Peter Davies at Albert Einstein College of Medicine.

### 4.2 Animals and treatment

Homozygous human P301S tau transgenic mice were generous gifts from Michael Goedert (Allen et al., 2002). One‐month‐old mice (male and female, *n* = 9 per group) were treated daily with curcumin analog C1 (5 and 10 mg per kg body weight) or vehicle for 3 months. To avoid possible injury to mice from long‐term oral gavage, compound C1 was mixed in regular feed according to the actual dosage used (5 and 10 mg/kg body weight, as low and high dosages, respectively). C1 was suspended well in distilled water and mixed with measured weight of regular feed powder. The mixture was kept at 55°C to evaporate excessive water. Fresh feed containing C1 was prepared twice weekly. The assessment of animals' body weight and calculation of feed consumption were performed every 2 weeks. Mice at 4 months of age after treatment were used for behavior tests and then sacrificed for biochemical analysis.

Male 5xFAD mice were purchased from Jackson Laboratory (Stock No: 006554) and maintained at 23 ± 2°C and 60 ± 15% relative humidity with free access to feed and water. 5xFAD male mice were inbred with wild‐type (WT, C57/BL6) female mice to produce heterozygous offspring. Offspring were genotyped to select 5xFAD (*APP* KM670/671Nl [Swedish], *APP* I716V [Florida], *APP* V717I [London], *PSEN1* M146L [A>C], *PSEN1* L286V) gene mutations mice. Two‐month‐old WT and 5xFAD mice (male and female, *n* = 10–14 per group) were treated daily with vehicle (1% sodium carbonyl methylcellulose [CMC‐Na; Sigma‐Aldrich, C5678]), curcumin analogue C1 (5 and 10 mg/kg), or curcumin (50 mg/kg) suspended in 1% CMC‐Na by oral gavage for 4 months.

Homozygous 3xTg mice (*APP* KM670/671Nl [Swedish], *MAPT* P301L, *PSEN1* M146V) were purchased from Jackson Laboratory (Stock No: 004807), produced in Hong Kong Baptist University animal house and maintained at 23 ± 2°C temperature, 60 ± 15% relative humidity with free access to feed and water. Seven‐month‐old WT (129/SvJ, *n* = 6, female) and 3xTg mice (*n* = 12, female) were treated daily with vehicle (1% CMC‐Na; Sigma‐Aldrich, C5678]), curcumin analogue C1 (5 and 10 mg/kg) or curcumin (50 mg/kg) suspended in 1% CMC‐Na by oral gavage for 7 months.

All animal care and experimental procedures were approved by the Hong Kong Baptist University Committee on the Use of Human and Animal Subjects in Teaching and Research.

### 4.3 Rotarod test

The limb motor coordination was analyzed by using Rotarod (Harvard apparatus, SeDaCom v2.0.000). Animals were introduced into the experimenting room 30 min before the start of experiment. Mice were trained for three consecutive days at constant speed 4, 8, and 12 RPM, respectively. Each day mice were trained for three times, and the time spent on rotor was recorded. On the 4th day in the acceleration speed mode (4–40 RPM in 5 min), maximum time spent on the rotor was recorded.

### 4.4 Contextual fear conditioning

Contextual fear conditioning (CFC) was used to evaluate the fear memory reconsolidation of 5xFAD mice after treatment. The experiment was conducted 30 min after the drug treatment in an ANY maze fear conditioning system under licensed ANY maze tracking system connected to a Windows XP computer. A digital camera was installed on each chamber, and signals were sent to the computer for analysis. The experiments were conducted in two sound‐proof chambers. All the experiments were conducted with continuous 40 units of white noise and 40 lux white light. During training, mice were allowed to explore the platform for 2.5 min and then received three repeated footshock cycles (30 s) at 30‐s intervals, each starting with a cue tone (28 s, 1,500 Hz) and ending with a foot shock (30.0 mA, 2 s). On the second day, the mice were allowed to explore the platform for 3 min followed by a cue tone (30 s, 1,500 Hz) without foot shock, and the freezing time was recorded. Contextual fear memory formation and the subsequent remote memory stabilization were evaluated by scoring freezing index (the absence of all but respiratory movement).

### 4.5 Open‐field test

3xTg mice were treated with the indicated compounds for 7 months, and then, the open‐field test was performed to evaluate the exploratory behavior in a novel environment. The experiment was conducted 30 min after the last dose was administered. Mice placed in the center of an empty open field (25 cm × 25 cm) were allowed to explore the environment for 5 min without any external disturbance. ethovision xt software was used to measure the patterns of movement, time spent in the center and margin, velocity and distance travelled; these measurements were used as indicators of agility and exploration.

### 4.6 Morris water maze

3xTg mice were treated with the indicated compounds for 7 months, and Morris water maze (MWM) was used to evaluate spatial learning memory. Morris water maze is a circular water (21 ± 1ºC) container, 1 m in diameter. Animals were prequarantined and acclimatized to the experimentation room at least 1 week before the start of the experiment. The experiment was started with a 1‐day visual platform trial. Each mouse underwent four visible platform trails (each trial 60 s) with different platform placement, positioned above the water level. Hidden platform training was conducted in water mixed with a nontoxic paint to make it opaque. Mice were trained for six continuous days, each day composed of different session with four hidden platform trials (each trial 60 s). Each session was designed with randomized animal introduction pattern into the water maze with unchanged platform position. Each trial was conducted with 30‐min inter‐trial interval and provided with external cues. To assess long‐term spatial memory retrieval, a probe trial (60 s) was conducted 24 hr after the last training session. For the probe trial, the water maze was divided into four quadrants. The hidden platform in the target quadrant was removed after recording its location. ethovision xt software was used to observe and record the swimming pattern of each mouse. During hidden platform training sessions, time spent to find the platform was taken as a parameter to evaluate memory acquisition. In the probe trial, the percentage of time spent in the target quadrant compared with the other three quadrants was calculated.

### 4.7 Electrophysiology for long‐term potentiation

#### 4.7.1 Animals and treatment

Three‐month‐old female c57BL/6 mice (*n* = 6) and heterozygous female 5xFAD mice were treated with vehicle (1% CMC‐Na; *n* = 6) or 10 mg/kg of C1 (*n* = 6) by oral administration for 3 months.

#### 4.7.2 Electrophysiological recordings with micro‐electrode array recording system

After decapitation, mouse brain was quickly dissected and placed in sucrose‐substituted ice‐cold artificial cerebrospinal fluid (sucrose‐aCSF, 120 mM sucrose, 10 mM d‐glucose, 2.5 mM KCl, 1.25 mM KH_2_PO_4_, 0.5 mM CaCl_2_, 10 mM MgSO_4_, 64 mM NaCl, and 26 mM NaHCO_3_, gas with 5% CO_2_/95% O_2_). Acute horizontal brain slices (350 µm) were sectioned with a vibrating microtome (5100mz; Campden Instruments) and recovered in the recording aCSF (recording aCSF, 10 mM d‐glucose, 3.5 mM KCl, 1.25 mM KH_2_PO_4_, 2.5 mM CaCl_2_, 1.5 mM MgSO_4_, 120 mM NaCl, and 26 mM NaHCO_3_, gas with 5% CO_2_/95% O_2_) at 32°C for 1 hr. The brain slices were then incubated in the oxygenated recording aCSF at room temperature before and during recording. To record field excitatory postsynaptic potential (fEPSP), brain slices were transferred to a 16 channel multi‐electrode array probe chamber (MED‐PG501A; Alpha MED Scientific). The slices were carefully relocated such that the multi‐electrode array was situated in the CA1 region of the hippocampal Schaffer Collateral/Commissural pathway. Oxygenated recording aCSF was perfused to the probe chamber at 2 ml/min. Bi‐phasic field pulses stimuli (10–60 µA, 0.2 ms) were delivered to Schaffer Collateral/Commissural pathway to invoke EPSP every 20 s, and slope of the fEPSP was recorded by MED64‐Quad II recoding system (Alpha MED Scientific). The intensity of the bi‐phasic field pulses stimuli for each brain slices was predetermined by the input/output (I/O) response curve. Stimuli that invoke 40% of maximum response amplitude were typically selected for each slice. The recordings were continued for 20 min to ensure a stable baseline response. Then, 5 trains of theta burst stimulation (TBS, 10 burst pulses at 100 Hz with 200‐ms interval for each pulse and 30‐s interval for each train) were delivered to the slice to induce long‐term potentiation (LTP). The increase in fEPSP slope was monitored for 60 min to ensure its stability.

### 4.8 Tissue extraction and Western blotting analysis

For P301S mice, half brain tissues were lysed with RIPA buffer (TBS with 1% NP‐40, 1% sodium deoxycholic acid, 0.1% SDS, and protease phosphatase inhibitor cocktails) and centrifuged at 21,130 *g* for 30 min to obtain the RIPA‐soluble fraction. Soluble and insoluble proteins were further extracted by sarkosyl fractioning according to the previous protocols (Polito et al., 2014). Briefly, the brain lysates were resuspended in 1% sarkosyl in TBS with protease inhibitor and phosphatase inhibitor cocktails, sonicated, incubated in shaking for 30 min, and ultracentrifuged at 100,000 *g* for 1 hr. The supernatants (sarkosyl‐soluble) were collected, and the pellets containing the sarkosyl‐insoluble material were resuspended in PBS. For 5xFAD mice, half brain tissues were lysed with RIPA buffer. For 3xTg mice, the hippocampi were dissected and lysed with RIPA buffer and the supernatant was collected. Protein concentration was estimated by BCA assay. Protein lysates were separated by 10%–15% SDS‐PAGE, transferred onto PVDF membrane, blocked with nonfat milk, and incubated overnight with primary antibodies and then with the respective secondary antibodies for 2 hr at room temperature. Protein chemiluminescence signal was detected by using ECL kits and quantified using imagej software.

### 4.9 Immunohistochemistry

Mice half brains were fixed with 4% paraformaldehyde in PBS overnight and dehydrated in 30% sucrose in PBS for at least 24 hr. Brain sections (30 µm) were cut on a microtome and stored at 4°C in PBS + 0.1% sodium azide. For fluorescence labeling, sections were permeabilized for 10 min with cold PBS containing 0.5% Triton X‐100 and blocked with 3% bovine serum albumin for 1 hr at room temperature. The sections were incubated with primary antibodies and Alexa Fluor conjugated secondary antibodies. After nuclear staining with DAPI, the slices were mounted with FluorSave reagent and visualized using the Eclipse 80i fluorescence microscope (Nikon Instruments Inc.). For DAB staining, the sections were incubated with biotinylated 4G8 antibody overnight and then incubated with Avidin‐Biotin enzyme complex (Vecstatin‐ELITE ABC‐HRP kit) and Aβ plaques were visualized by DAB peroxidase and quantified using imagej software.

### 4.10 ELISA assay of Aβ_1–40_ and Aβ_1–42_

The whole‐brain samples were sequentially extracted through a three‐step extraction procedure (Youmans et al., 2011) with minor modifications. Mouse brain samples were homogenized in 1/10 volumes of TBS (containing protease inhibitor, phosphatase inhibitor, and PMSF) and centrifuged in Beckman Coulter Optima™ L‐80XP Ultracentrifuge at 100,000 *g*, 4°C for 1 hr. TBS supernatant was fraction collected, and the pellets were resuspended in 1% Triton X and proceeded for ultracentrifugation. The resulting Triton soluble fraction was collected, and the pellets were homogenized again in 70% formic acid (FA) in 50 mM TBS (pH = 7.4). After brief sonication, samples were centrifuged at 20,000 *g*, 4°C for 20 min. FA fractions were neutralized with Tris buffer (pH = 11) at 1:12 dilution rate. Aβ in TBS‐Triton fraction was regarded as the detergent‐soluble, while Aβ in neutralized formic acid fraction was considered as the detergent‐insoluble fraction and subjected for sandwich ELISA. The ELISA plates (Thermo Fisher Scientific; 439454) were coated overnight with 6E10 (4 µg/ml) antibody in phosphate well coating buffer (pH = 8.3) and blocked with 4% block ace in PBS for 2 hr. Equal amounts of the FA‐neutralized fraction and equalized protein concentration of TBS‐Triton fractions were loaded in duplicate wells and incubated at room temperature for 2 hr under shaking. Biotinylated 5C3 (Nanotools, 0060S) and 8G7 (Nanotools, 0061S) were used to determine Aβ_1–40_ and Aβ_1–42_, respectively. Secondary antibodies were diluted at 1:1,000 concentration in 1% Block ace solution and incubated at room temperature for 2 hr. After washing the plates with PBST, streptavidin HRP (Dako, P03971) was added, and the plates were incubated at 37°C for 1 hr. TMB substrate (BD Biosciences, 555214) was added to the plates, and they were incubated at room temperature for 30 min. Finally, an equal volume of 2 M H_2_SO_4_ was added, and absorbance was measured at 450 nm. Synthetic Aβ peptide (AnaSpec, AS‐20276 and AS‐24236) was used to construct each standard curve.

### 4.11 In vitro studies

N2a cells were cultured in DMEM/Opti‐MEM (50:50) supplemented with 2% FBS. The cells were transiently transfected with human APP^Swe/Ind^ (APP 695 Swedish/Indiana mutation, Young‐Pearse et al., 2007, 30145; Addgene) or P301L‐Tau (Hoover et al., 2010, 46908; Addgene) plasmids with empty vectors as the control and then treated with C1 for 16 hr or EBSS for 4 hr. For cycloheximide (CHX) chase assay, N2a‐APP cells were pretreated with C1 and then treated with 50 μg/ml CHX followed by collecting cells at several time intervals. Bafilomycin A1 (1334; Tocris Bioscience; 100 nM) was used to inhibit lysosome function as previously described (Xiao et al., 2015). For TFEB knockdown, N2a cells were infected with lentivirus expressing nontarget shRNA (Sigma‐Aldrich; SHC002V) or mouse *Tfeb* shRNA (Sigma‐Aldrich; TRCN0000085548) for 48 hr and then incubated with medium containing puromycin (1.5 μg/ml) until resistant colonies were identified.

### 4.12 Statistical analysis

All data are presented as mean ± *SEM*. One‐way analysis of variance (ANOVA) followed by the Dunnett's multiple comparison test, or two‐way ANOVA followed by Bonferroni post hoc test was performed using the GraphPad Prism 8.0.1. Outliers were identified using ROUT method with *Q* = 1% using GraphPad Prism 8.0.1. A probability value of *p* < .05, *p* < .01, and *p* < .001 was considered to be statistically significant.

## CONFLICT OF INTEREST

The authors declare that they have no conflict of interest.

## AUTHOR CONTRIBUTIONS

JXS, SM, YZ, SSKD, CBY, SGS, ZZ, AI, BCKT, KHC, JHL, and WBS performed experiments and analyzed the data; JXS, KHC, and ML designed the study; SSKD provided the P301S, 5xFAD, and 3xTg mice; CT and NX provided funding support and overall guidance. All authors made comments on the manuscript.
