# Supplementary material for: A small molecule transcription factor EB activator ameliorates beta‐amyloid precursor protein and Tau pathology in Alzheimer's disease models
Source: Aging Cell. 2019 Dec 19;19(2):e13069. doi: 10.1111/acel.13069 (PMC6996953; doi:10.1111/acel.13069)
Supplement: Supplementary file 1 [file ACEL-19-e13069-s001.pdf]

SONG ET AL., SUPPLEMENTAL INFORMATION

SUPPORTING INFORMATION LISTING

Table S1. Two-Way ANOVA analysis of sex-drug interaction in 5xFAD mice

Fig. S1. The neuroprotective effects of compound C1 in P301S mice.

Fig. S2. The neuroprotective effects of compound C1 in 5xFAD mice and the brain concentrations of C1.

Fig. S3. Effects of compound C1 and curcumin on MTORC1 activity in the hippocampus of female 3xTg mice brain.

Fig. S4. Effects of compound C1 on endogenous APP and Tau.

Fig. S5. Effects of compound C1 on GSK3B and AKT.

Table S1. Two-Way ANOVA analysis of sex-drug interaction in 5xFAD mice

	**Interaction**		**Drug**		**Sex**	
	P	F (DFn, DFd)	*P*	F (DFn, DFd)	*P*	F (DFn, DFd)
WB-Fl-APP	0.1378	F (3, 40) = 1.945	0.2034	F (3, 40) = 1.605	0.0003	F (1, 40) = 15.280
WB-CTF-β	**0.0025**	F (3, 40) = 5.679	0.0006	F (3, 40) = 7.136	0.0066	F (1, 40) = 8.227
WB-CTF-α	**0.0002**	F (3, 40) = 8.392	0.0002	F (3, 40) = 8.495	0.0007	F (1, 40) = 13.400
WB-Aβ	0.1678	F (3, 40) = 1.773	<0.0001	F (3, 40) = 15.910	0.0019	F (1, 40) = 11.060
ELISA-Aβ42	0.5875	F (3, 38) = 0.651	<0.0001	F (3, 38) = 18.150	0.0088	F (1, 38) = 7.625
ELISA-Aβ40	0.6317	F (3, 37) = 0.580	<0.0001	F (3, 37) = 9.387	0.0031	F (1, 37) = 10.030
IHC-Aβ load	0.8302	F (2, 30) = 0.187	<0.0001	F (2, 30) = 18.140	0.8987	F (1, 30) = 0.016
WB-m-CTSD	0.1502	F (3, 40) = 1.870	<0.0001	F (3, 40) = 9.456	0.0638	F (1, 40) = 3.635
WB-LAMP1	0.8890	F (3, 40) = 0.211	<0.0001	F (3, 40) = 9.320	0.2318	F (1, 40) = 1.474
WB-nTFEB	0.9084	F (3, 40) = 0.181	0.0033	F (3, 40) = 5.391	0.8257	F (1, 40) = 0.0492
WB-LC3B-II	0.3085	F (3, 40) = 1.238	<0.0001	F (3, 40) = 9.789	0.7582	F (1, 40) = 0.0961

Figure S1

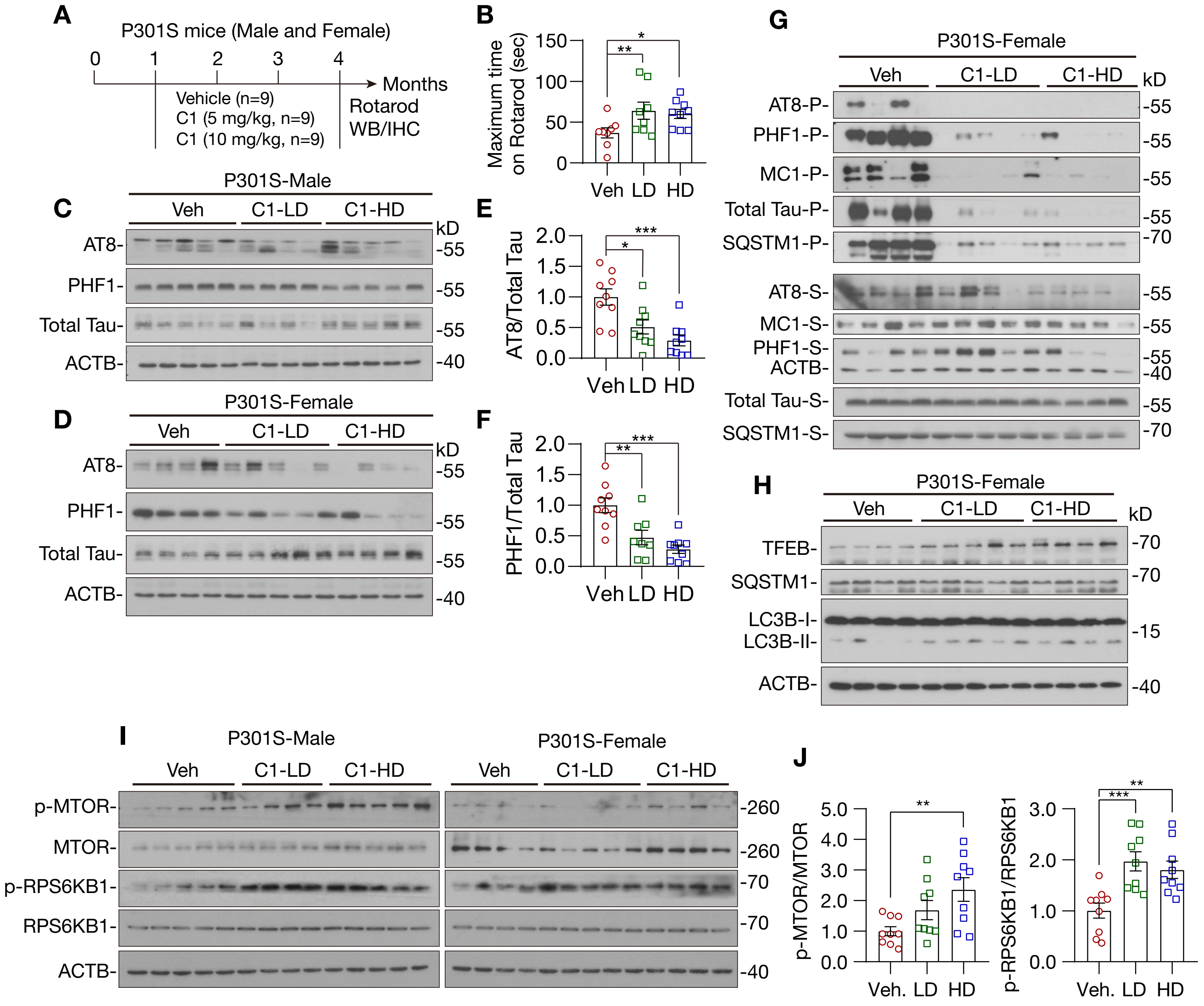
A
P301S mice (Male and Female)
0 1 2 3 4
Months
Vehicle (n=9)
C1 (5 mg/kg, n=9)
C1 (10 mg/kg, n=9)
Rotarod
WB/IHC
B
Maximum time on Rotarod (sec)
Veh LD HD
C
P301S-Male
Veh C1-LD C1-HD
AT8-
PHF1-
Total Tau-
ACTB-
D
P301S-Female
E
AT8/Total Tau
F
PHF1/Total Tau
G
AT8-P-
PHF1-P-
MC1-P-
Total Tau-P-
SQSTM1-P-
AT8-S-
MC1-S-
PHF1-S-
Total Tau-S-
SQSTM1-S-
H
TFEB-
SQSTM1-
LC3B-I-
LC3B-II-
I
p-MTOR-
MTOR-
p-RPS6KB1-
RPS6KB1-
J
p-MTOR/MTOR
p-RPS6KB1/RPS6KB1
Veh. LD HD

Fig. S1. The neuroprotective effects of compound C1 in P301S mice. (**A**) Experimental protocols for P301S mice. (**B**) Rotarod test. All the values from both male and female mice (n = 9) were quantified as average ± s.e.m. *P < 0.05 and **P < 0.01 vs. vehicle group (Veh.) respectively analyzed by one-way ANOVA. LD = low dose, 5 mg/kg; HD = high dose, 10 mg/kg. (**C-D**) Western blots of AT8 (S202/T205), PHF1 (S396/S404) and total Tau in the RIPA-soluble brain lysates from male (**C**) and female (**D**) P301S mice. (**E-F**) Quantification of AT8/total Tau (**E**) and PHF1/total Tau (**F**). All the values from both male and female mice (n = 9) were quantified as average ± s.e.m. *P < 0.05, **P < 0.01 and ****P < 0.0001 vs. Veh. group respectively analyzed by One-way ANOVA. (**G**) Western blots of AT8 (S202/T205), PHF1 (S396/S404) and total Tau in the sarkosyl-soluble (S) and sarkosyl-insoluble (P) fractions from female P301S mice. (**H**) Western blots of TFEB, SQSTM1 and LC3B in the RIPA-soluble brain lysates from female P301S mice. (**I**) Western blots show the levels of phosphorylated (p-) and total MTOR (Ser2448) and RPS6KB1/p70S6K in P301S mice brains. (**J**) All the values from both male and female mice were quantified as average ± s.e.m. (n = 9). **P < 0.01 and ***P < 0.001 vs. Veh. group respectively analyzed by One-way ANOVA.

Figure S2

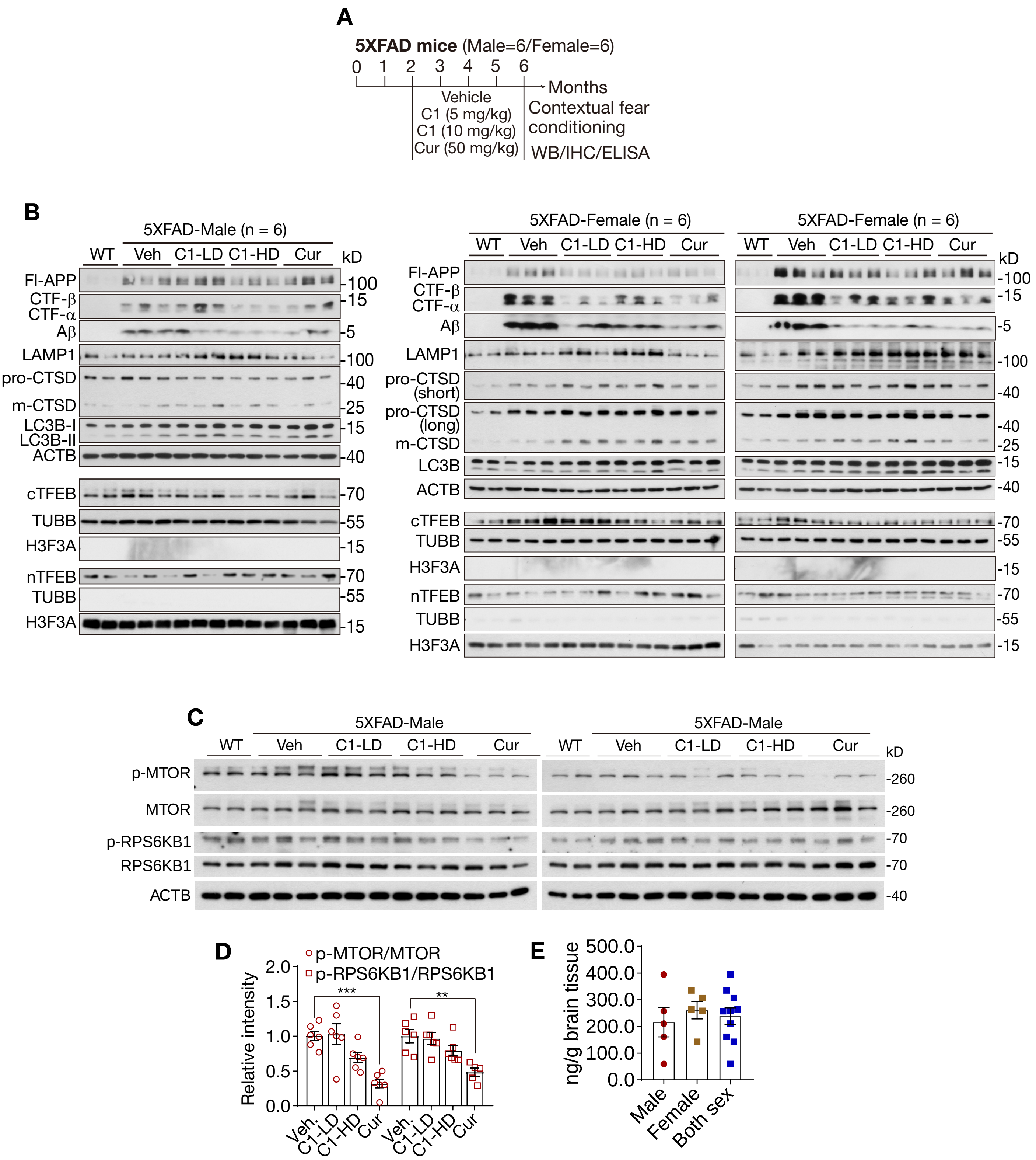
A
5XFAD mice (Male=6/Female=6)
0 1 2 3 4 5 6
Months
Vehicle
C1 (5 mg/kg)
C1 (10 mg/kg)
Cur (50 mg/kg)
Contextual fear conditioning
WB/IHC/ELISA
B
5XFAD-Male (n = 6)
5XFAD-Female (n = 6)
WT Veh C1-LD C1-HD Cur
kD
Fl-APP
CTF-β
CTF-α
Aβ
LAMP1
pro-CTSD
m-CTSD
LC3B-I
LC3B-II
ACTB
cTFEB
TUBB
H3F3A
nTFEB
pro-CTSD (short)
pro-CTSD (long)
LC3B
C
5XFAD-Male
p-MTOR
MTOR
p-RPS6KB1
RPS6KB1
D
p-MTOR/MTOR
p-RPS6KB1/RPS6KB1
Relative intensity
***
**
Veh. C1-LD C1-HD Cur
E
ng/g brain tissue
Male Female Both sex

Fig. S2. The neuroprotective effects of compound C1 in 5xFAD mice and the brain concentrations of C1. (**A**) Experimental protocols for 5XFAD mice. (**B**) Additional blots show the levels of full-length APP (Fl-APP), CTF-β/α, Aβ, LAMP1, CTSD, cytosolic and nuclear TFEB (cTFEB and nTFEB) and LC3B in the brain lysates of male and female mice. (**C**) Curcumin, but not C1 inhibits MTORC1 activity in 5xFAD mice brains. Western blots show the levels of p-/total MTOR and RPS6KB1/p70S6K in the brain lysates of 5xFAD mice treated with C1 and curcumin. (**D**) Data were quantified as average ± s.e.m. (n = 6). $^{**}P < 0.01$ and $^{***}P < 0.001$ vs. Veh. group respectively analyzed by One-way ANOVA. (**E**) The brain concentrations of C1 in male (n = 5) and female (n = 5) 5xFAD mice treated with 10 mg/kg of C1 for 4 months determined by LC-MS. Data was presented as mean ± s.e.m.

Figure S3

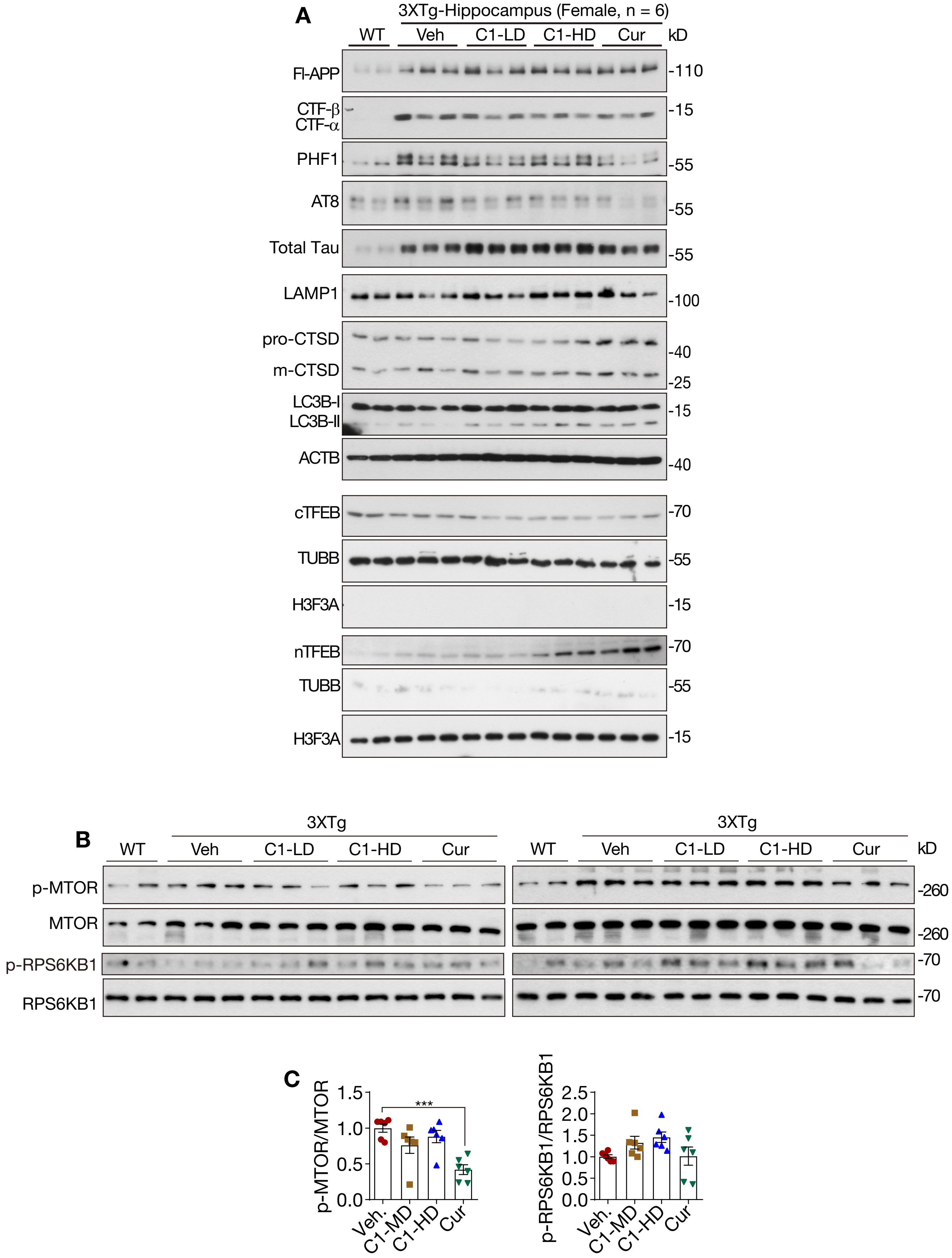
A
3XTg-Hippocampus (Female, n = 6)
WT
Veh
C1-LD
C1-HD
Cur
kD
Fl-APP
CTF-β
CTF-α
PHF1
AT8
Total Tau
LAMP1
pro-CTSD
m-CTSD
LC3B-I
LC3B-II
ACTB
cTFEB
TUBB
H3F3A
nTFEB
TUBB
H3F3A
-110
-15
-55
-55
-55
-100
-40
-25
-15
-40
-70
-55
-15
-70
-55
-15
B
3XTg
WT
Veh
C1-LD
C1-HD
Cur
kD
p-MTOR
MTOR
p-RPS6KB1
RPS6KB1
-260
-260
-70
-70
C
p-MTOR/MTOR
1.5
1.0
0.5
0.0
***
Veh.
C1-MD
C1-HD
Cur
p-RPS6KB1/RPS6KB1
2.5
2.0
1.5
1.0
0.5
0.0
Veh.
C1-MD
C1-HD
Cur

Fig. S3. Effects of compound C1 and curcumin on MTORC1 activity in the hippocampus of female 3xTg mice brain. (**A**) Another batch of blots showed the levels of Fl-APP, CTF-β/α, phosphorylated Tau (PHF-1, AT-8), total Tau, LAMP1, CTSD, cytosolic and nuclear TFEB (cTFEB and nTFEB) and LC3B. (**B**) Western blots showed the levels of p-/total MTOR and RPS6KB1/p70S6K. (**C**) All the values from female (n = 6) mice were quantified as average ± s.e.m. ***$P < 0.001$ vs. Veh. group analyzed by One-way ANOVA.

Figure S4

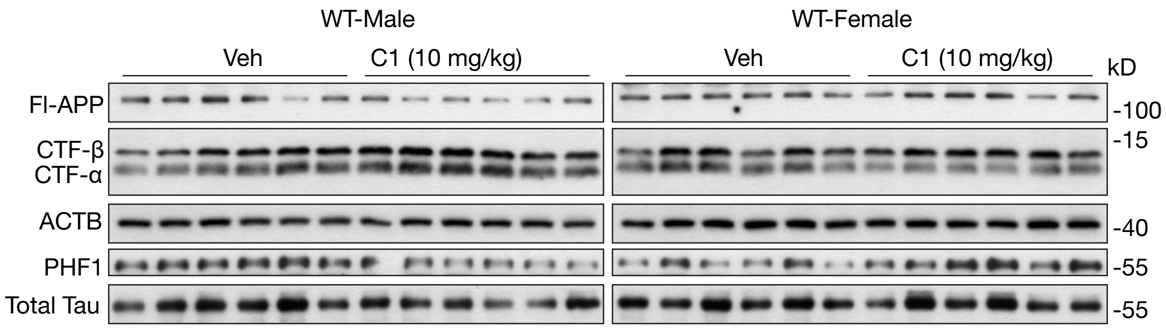

Fig. S4. Effects of compound C1 on endogenous APP and Tau. Wild type (WT) mice (6-month old, n = 6) were orally treated with vehicle (Veh) or C1 (10 mg/kg) for 7 months. Representative Western blots show the levels of Fl-APP, CTF-α/β, PHF1 and total Tau in the hippocampus. No significant difference between Veh. and C1 was observed and the quantification data was not shown.

Figure S5

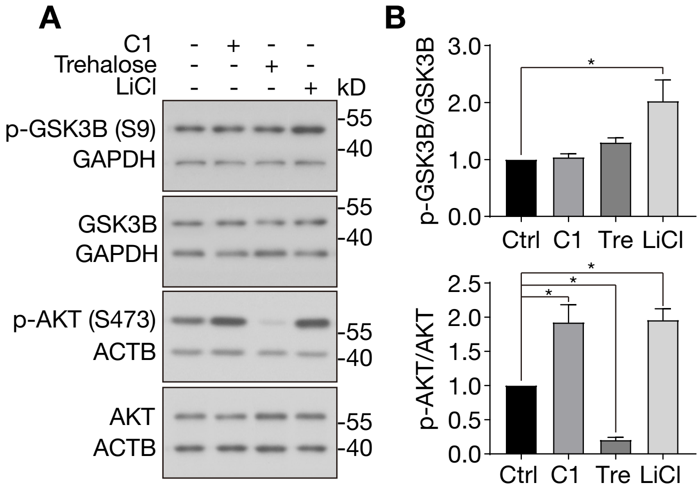

Fig. S5. Effects of compound C1 on GSK3B and AKT. N2a cells were treated with C1 (1 μM), trehalose (Tre, 100 mM) or LiCl (20 mM) for 24 h. (**A**) Representative Western blots show the levels of p-GSK3B (S9), total GSK3B, p-AKT (S473) and total AKT, with GAPDH or ACTB as loading controls. (**B**) Data are presented as the average ± s.e.m. from 2 independent experiments. *P < 0.05 vs. control, analyzed by One-way ANOVA.
